# Adjunct Strategies for Tuberculosis Vaccines: Modulating Key Immune Cell Regulatory Mechanisms to Potentiate Vaccination

**DOI:** 10.3389/fimmu.2016.00577

**Published:** 2016-12-16

**Authors:** Lakshmi Jayashankar, Richard Hafner

**Affiliations:** ^1^Columbus Technologies, Inc., Contractor to the National Institute of Allergy and Infectious Diseases, National Institutes of Health, Bethesda, MD, USA; ^2^Division of AIDS, National Institute of Allergy and Infectious Diseases, National Institutes of Health, Bethesda, MD, USA

**Keywords:** tuberculosis, vaccines, immunometabolism, immuno-oncology

## Abstract

Tuberculosis (TB) remains a global health threat of alarming proportions, resulting in 1.5 million deaths worldwide. The only available licensed vaccine, Bacillus Calmette–Guérin, does not confer lifelong protection against active TB. To date, development of an effective vaccine against TB has proven to be elusive, and devising newer approaches for improved vaccination outcomes is an essential goal. Insights gained over the last several years have revealed multiple mechanisms of immune manipulation by *Mycobacterium tuberculosis* (Mtb) in infected macrophages and dendritic cells that support disease progression and block development of protective immunity. This review provides an assessment of the known immunoregulatory mechanisms altered by Mtb, and how new interventions may reverse these effects. Examples include blocking of inhibitory immune cell coreceptor checkpoints (e.g., programed death-1). Conversely, immune mechanisms that strengthen immune cell effector functions may be enhanced by interventions, including stimulatory immune cell coreceptors (e.g., OX40). Modification of the activity of key cell “immunometabolism” signaling pathway molecules, including mechanistic target of rapamycin, glycogen synthase kinase-3β, wnt/β-catenin, adenosine monophosophate-activated protein kinase, and sirtuins, related epigenetic changes, and preventing induction of immune regulatory cells (e.g., regulatory T cells, myeloid-derived suppressor cells) are powerful new approaches to improve vaccine responses. Interventions to favorably modulate these components have been studied primarily in oncology to induce efficient antitumor immune responses, often by potentiation of cancer vaccines. These agents include antibodies and a rapidly increasing number of small molecule drug classes that have contributed to the dramatic immune-based advances in treatment of cancer and other diseases. Because immune responses to malignancies and to Mtb share many similar mechanisms, studies to improve TB vaccine responses using interventions based on “immuno-oncology” are needed to guide possible repurposing. Understanding the regulation of immune cell functions appropriated by Mtb to promote the imbalance between protective and pathogenic immune responses may guide the development of innovative drug-based adjunct approaches to substantially enhance the clinical efficacy of TB vaccines.

## Introduction

Tuberculosis (TB) caused by the bacillus *Mycobacterium tuberculosis* (Mtb), is the most common infectious cause of death worldwide. In 2014, an estimated 1.5 million deaths were attributed to TB (1). Approximately one-third of the world’s population is infected with Mtb, and 90–95% of those will remain latently infected and asymptomatic. The other 5–10% will progress to active disease (2). Bacillus Calmette–Guérin (BCG) vaccine has been available for more than 90 years but is not sufficiently successful in preventing active TB (3). The efficacy of BCG vaccine efficacy in preventing pulmonary TB is limited, with studies showing 0–80% protective benefit (4). BCG is 70–80% efficacious against severe forms of TB in childhood, particularly in infant meningitis (5). The variations in BCG vaccine efficacy found in different studies have been attributed to geographical differences, exposure to certain endemic mycobacteria, and varied manufacturing facilities with inconsistent quality control (4, 6). The tremendous variability associated with its protective effects against TB, specifically the waning of protection in the adolescent and adult populations has resulted in intense efforts to improve its partial efficacy (7). Currently, more than a dozen vaccine candidates for TB are at different stages of clinical trial development. Recent developments of prime-boost TB candidate vaccines include subunit, multi-epitopic candidate vaccines with potentially broad immunological coverage consisting of Mtb-secreted components (ESAT-6, the antigens 85A and 85B) and the PPE family members. Newer multistage subunit vaccine strategies have included antigens from dormancy/starvation, early reactivating (resuscitation-promoting factors), and active bacilli stages (8). BCG replacement vaccines include live recombinant BCG, modified non-pathogenic mycobacteria (*M. vaccae*, RUTI, and *M. smegmatis*), and inactivated whole cell vaccine (WCV) candidates (9–11). Although successful in animal models, the viral vectored MVA85A, the first candidate TB vaccine to enter efficacy trials showed disappointing results in Phase II trials (12, 13). The protein/adjuvant candidates including M72/AS01 and H4 and H56/IC31 have shown promising immunogenicity in Phase 1/II trials and are currently in efficacy trials with results awaited (14–16). The lack of known correlates of protection associated with different stages of TB infection and disease has been a barrier to the clinical evaluation of vaccine candidates, and the TB vaccine field is now redirecting its attention to basic discovery and preclinical development (17).

New strategies are needed to improve vaccine efficacy based on both a better understanding of the mechanisms mediating protective immunity and mechanism of subversion of host immune responses by immunopathogenic components of Mtb. Opinion on the balance between protective and destructive responses to Mtb infection in humans and animal models and the functional role of granuloma is still evolving (18, 19). A better understanding of the features that contribute to protective host responses to Mtb infection will aid in the identification of drugs that can be repurposed for use to potentiate current TB vaccination strategies. The Mtb-infected host-cell microenvironment is characterized by dysregulated immunoregulation and immunometabolism signaling pathways, for example, Th1/Th17 versus Th2 balance, regulatory T (Treg) and suppressive myeloid cell populations, T cell anergy, and a shift from M1 to M2 polarized macrophages (20–24).

Immunologic responses to malignancies and TB have many similarities in terms of persistent inflammation and innate and T cell-mediated responses (20). In this review, some of the key pathways regulating the function of Mtb-infected antigen-presenting cells (APC) are explored. Improved understanding of the fundamental mechanisms of immune cell suppression and downregulation of protective immune responses by Mtb may lead to the introduction of novel interventions to promote vaccine-induced anti-Mtb immunity. The relevant immune cellular pathways are well known in various other diseases, but a cohesive picture of the affected immunoregulatory and immunometabolism machinery is yet to be established for TB vaccinology.

Potential adaption of the following two key strategies will be delineated:

1. Immuno-oncology: immune cell coreceptor checkpoints that determine the intensity of T-cell responses to particular antigens and imbalances in the numbers and activity of immune regulatory cells.
2. Immunometabolism: a relatively new concept developed in oncology, which refers to manipulation of checkpoints causing changes in cell metabolism that are required for and/or direct immune cell differentiation and function.

This review will focus on the potential of these interventions to be tested as adjunct strategies to shift the balance of TB vaccines toward protective responses and potentiate host responses induced by vaccines.

## Targeting Cell Regulatory Pathways and Immune Checkpoints as Adjunct Strategies for TB Vaccination

### Targeting Autophagy Regulation Pathways

Autophagy is a process for maintaining cellular homeostasis, particularly during stress conditions, by continual degradation of damaged organelles, protein aggregates, and intracellular pathogens through the autophagosome. Autophagic processes also contribute to the activation of innate and adaptive immune responses (25–27). During Mtb infection, pro-inflammatory cytokines positively regulated by autophagy induction control intracellular Mtb proliferation, and both TLR (TLR2, TLR4, and TLR9) and non-TLR pathways are triggered to degrade the bacteria (28–35). Nevertheless, Mtb in autophagosomes can escape elimination by autophagy by preventing lysosomal fusion and acidification though the underlying mechanisms of escape remain poorly understood (27, 36–38).

Experimental evidence suggests that vaccine efficacy can be improved through enhanced antigen presentation to T cells by boosting autophagy-mediated antigen presentation (39–41). Inhibition of the mechanistic target of rapamycin (mTOR) is a well-known mechanism for inducing autophagy (Table 1). Rapamycin (sirolimus) is a well-characterized inhibitor of mTOR (33, 42). The efficacy of BCG vaccine is improved in a murine model, and antigen presentation by DCs *in vitro* is enhanced by rapamycin with an increase in Th1 responses (39, 43). As inhaled drug and vaccine delivery platforms gain recognition and prolonged use of autophagy inducers can result in immunosuppression, rapamycin delivered in poly lactide-co-glycolide nano particles can maintain high localized intracellular drug concentration and minimize systemic side effects (40, 44). Coadministration of a DNA vaccine composed of the immunodominant mycobacterial antigen Ag85B and incorporating an autophagy-inducing mTOR-KD plasmid induced primarily a Th1 immune response (33, 39). This DNA vaccine was delivered by chitosan particles to enhance mucosal immunity.

**Table 1:** **Agents that target immune checkpoints, immune regulatory cells, and key pathways involved in Mtb pathogenesis**.

Target	Drug examples	Probable therapeutic mechanism	Preclinical studies	Preclinical studies in TB vaccination
**Autophagy modulation**				
Direct mechanistic target of rapamycin (mTOR) inhibitors	Sirolimus, everolimus,^a^ ridaforolimus^a^	Reversal of mTOR-mediated inhibition of autophagy	TB infection and cancer models	Improved Bacillus Calmette–Guérin (BCG) vaccine efficacy
mTOR-independent agents	Imatinib/other tyrosine kinase inhibitors (TKIs), metformin (MET), and carbamazepine	Increase autophagy: improve pathogen killing and processing of antigenic material for T-cell presentation	TB infection and cancer models	ND
**Immune checkpoint modulation**				
Programed death-1/CTLA-1 inhibitors	Ipilimumab, pembrolizumab, and nivolumab	Checkpoint blockade removes inhibitory coreceptor initiated signaling (including reversal of T cell exhaustion)	TB infection and cancer models	ND
OX40 stimulator	OX40 agonist monoclonal antibody	Expansion of effector T cells and generation of memory T cells	Cancer models	Improved BCG vaccine efficacy
A2A coreceptor inhibitor	Istradefylline^b^	Reversal of T cell exhaustion	Cancer models, Parkinson’s disease	ND
**Modulation of immune regulatory cells: Tregs and myeloid-derived suppressor cells**				
Damage-associated molecular patterns inhibitor	Tasquinimod	Inhibition of induction/activity of regulatory/suppressor cells	Cancer models	ND
Indoleamine 2,3-dioxygenase inhibitor	Epacadostat	Same	TB infection	ND
Tyrosine kinase inhibitors	Sunitinib, dasatinib, imatinib	Same	TB infection (for imatinib)	ND
PDE5 inhibitors	Sildenafil^c^	Same	Cancer models	ND
Fatty acid oxidation inhibitor	Etomoxir^d^	Same	Cancer models	ND
Arginase inhibitor	Nor-NOHA^e^	Same	Cancer models	ND
**Immunometabolism regulation**				
Adenosine monophosophate-activated protein kinase activators	MET, AICAR^f^	Anti-inflammatory, increase autophagy, and improve DC, TH1 CD4 cell, and CD8 memory cell development	TB infection and cancer models	ND
Sirtuins				
Activators	Resveratrol, pterostilbene,^g^ Sirt1 activator compounds in development	Anti-inflammatory and increase autophagy	Cancer models and viral infections	ND
Inhibitors	Sirtinol, cambinol^h^	Increase Th1/Treg ratio	Cancer models and viral infections	ND
**Core signaling pathway modulators**				
Wnt inhibitors	Tankyrase inhibitors, flavonoids, monensin, and resveratrol	Reversal of activation of multiple inhibitory signaling pathways	TB infection and cancer models	ND
Notch inhibitors (gamma-secretase inhibitors)	RO4929097^i^	Same	TB infection and cancer models	ND
Sonic hedgehog inhibitors	Vismodegib, sonidegib	Same	TB infection and cancer models	ND
Glycogen synthase kinase-3β inhibitors	Tideglusib	Same	TB infection and cancer models	ND
**Epi-drugs**				
Histone deacetylase inhibitors	Sodium butyrate	Histone deacetylation, inducing transcription of, e.g., antimicrobial peptides	Cancer models	ND

*ND, not done*.

*References for table not provided in the text: ^a^Huang et al. (100); ^b^Leone et al. (101), Muller (102); ^c^Serafini et al. (103), Weed et al. (104); ^d^Byersdorfer (105); ^e^Rodriguez and Ochoa (106); ^f^Yang et al. (107); ^g^Kala et al. (108); ^h^Hu et al. (109); and ^i^Xu et al. (110)*.

Inducing autophagy by mTOR-independent methods is also being pursued. Microtubule-associated protein light chain-3 (LC3) transports Mtb lipoprotein (LpqH) to autophagosomes and is involved in autophagy activation *via* TLR1/2/CD14 receptors. A LC3–LpqH DNA vaccine enhanced protective efficacy against Mtb in mice (45). Screening of FDA-approved drugs has identified compounds stimulating autophagic killing of mycobacteria at therapeutic concentrations though none of these agents have been tested preclinically in conjunction with TB vaccine candidates. Carbamazepine triggers autophagy independently of mTOR and effectively targets multidrug-resistant Mtb *in vivo* by stimulating both innate and adaptive immunity (46). Regulation of the acidification of intracellular compartments has also shown potential to enhance host defense against pathogens. Imatinib, a tyrosine kinase inhibitor (TKI), promotes lysosome acidification and anti-mycobacterial activity in macrophages in a mouse model, and is being further tested as a potential complementary therapy for TB (47–49). TKI inhibition is a strategy for improving autophagy but may also have other mechanisms of action, including suppression of myeloid-derived suppressor cells (MDSCs) and Tregs. Statins, HMG-CoA reductase inhibitors, and autophagy-inducing agents have shown immunomodulatory properties in TB models, resulting in increased bacterial clearance and improved efficacy of first-line TB drugs with a substantial decrease in inflammation (Table 1) (50, 51).

### Immune Checkpoint Modulation

#### Modulation of Programed Death-1 Pathway

The programed death-1 (PD-1) receptor is a member of the CD28 superfamily that negatively regulates T cell responses to antigen stimulation and enhances tolerance, triggered by interaction with its ligands PD-L1 and PD-L2 (52, 53). PD-1 is expressed on Tregs, natural killer (NK) cells, follicular T and B cells, and APCs (53). PD-1 has recently emerged as an important co-inhibitory molecule and “immune checkpoint” in a number of chronic intracellular infections, including TB, and overexpression of PD-1, cytotoxic T-lymphocyte antigen (CTLA-4) receptor, and TIM3 has been associated with T-cell exhaustion (54–56). In human TB infection, PD-1 activity induces Tregs and immunomosuppressive cytokines such as interleukin (IL)-10 and TGF-β (57, 58). HIV–TB coinfection is associated with increased expression of PD-1 on Mtb-specific cytokine-secreting CD4+ cell subsets (59).

Checkpoint blockade, i.e., removal of inhibitory coreceptor initiated signaling, is a strategy used successfully in cancer treatment for vaccine potentiation. Monoclonal antibodies (mAbs) that modulate inhibitor signaling by CTLA-4 and PD-1, e.g., ipilimumab, pembrolizumab, and nivolumab, are now in wide clinical use for several cancers (Table 1) (60). Combination of checkpoint blockade with PD-1 and CTLA-4 inhibition enhances response rates in patients with minimal responses to single checkpoint blockade with lesser toxicity compared to previous immunotherapies, e.g., IL-2 (60, 61). PD-1 blockade with the cancer vaccine TEGVAX in mice showed regression of established tumors, providing a rationale using vaccines combined with immune checkpoint blockade (62).

The overall role of PD-1 blockade in TB remains a complicated issue. PD-1-deficient mice have shown dramatically reduced survival compared with wild-type mice post-Mtb infection. Increased levels of pro-inflammatory cytokines and uncontrolled bacterial proliferation were seen in the lungs of these mice (63). These observations indicate that control of Mtb infection requires a carefully balanced immune response that contains the infection without causing pathology (64).

Interestingly, the specific role of PD1–L2 is currently of interest as a therapy for malaria. Blockade of PD1–L1 binding resulted in unfavorable outcome in a malaria mouse model, but blockade of PD1–L2 did not, suggesting different functions of the two PD-1 ligand functions in malarial infections (65). Administration of soluble multimeric PD-L2 outcompeted PD-L1 for PD-1 binding and improved CD4+ T cell responses in mice with lethal malaria, dramatically improving survival (66). The results also suggest that PD-L2 not only competes with PD-L1 but also has a stimulatory action *via* interaction with a different coreceptor.

Additional studies are needed to further investigate these issues that might allow tuning of TB vaccine responses to be more effective. Energy supply insufficiency due to cell metabolic alterations directed by PD-1 signaling leads to exhaustion and begins early in CD8+ T cell responses in chronic lymphocytic choriomeningitis virus infection (67). Downregulation of the glycolytic and peroxisome proliferator-activated receptor c coactivator 1α (PGC-1a) pathways leads to reduced mitochondrial energy availability. Additionally, mTOR signaling drives anabolic metabolism of effector T cells, exacerbating energy deficiency, further contributing to cell exhaustion. The reversion of these changes in a subset of exhausted CD8+ T cells by anti-PD-1 therapy suggests that targeted agents to directly inhibit these metabolic changes should be explored (67). The nuclear factor of activated T cells (NFAT) proteins is also primary in the establishment of T cell exhaustion in mouse models of tumor growth and bacterial infection (68). During persistent antigen stimulation, NFAT promotes T cell exhaustion by binding directly to regulatory regions of PD-1 and TIM3 (68). Targeting NFAT isoforms or their associated signaling molecules is being actively pursued in cancer therapy (69).

#### Stimulation through Coreceptors: OX40

The various checkpoint co-inhibitors may need to be combined with T-cell co-stimulators (or stimulators could be used alone), for effective CD4+ Th1 and CD8+ T-cell development and subsequent sustained T-cell memory responses (70). CD134 (OX40), a TNF-α receptor superfamily member, is expressed transiently on newly activated T cells and NK cells (71, 72). CD134–CD134 ligand interactions are crucial for the expansion of effector T cells and generation of memory T cells (73). OX40 agonist mAb administration results in a Th1-cytokine-driven response enhancing IL-2 production by effector T cells and preventing the expression of CTLA-4, FoxP3, and IL-10 (71, 74). Compared with BCG vaccination alone, OX40 ligation with BCG vaccination in a murine model provided enhanced protection against aerosol and intravenous Mtb challenge with NK cells playing a crucial role (71). In cancer therapy, the beneficial results of combination immunotherapy targeting both costimulatory and co-inhibitory molecules (combined OX40/PD-1 or OX40/CTLA-4 mAb therapy) dramatically improved survival in the prostate, ovarian carcinoma, and sarcoma models (70, 75). The adjunct potential of OX40 agonists, in conjunction with CTLA–4 mAb, has been shown for infection by the intracellular bacterium, *Leishmania donovani* (76). The role of activation of stimulatory coreceptors to improve vaccine responses deserves further investigation.

### Modulation of Immune Regulatory Cells

#### Regulatory T Cells

Th1-type immunity is downregulated by a subset of immunosuppressive CD4+ T cells expressing CD25 and the transcription factor FoxP3, referred to as Tregs (77–79). As excessive inflammation related to highly activated populations of CD4+ T cells can cause tissue damage and exacerbate TB disease, a critical balance of regulatory and effector T-cell responses govern the outcome of infection (80, 81). Tregs suppress both T cells and APCs, thus enhancing persistent infection (79, 82–84). Expansion of Tregs in active TB has been observed both at organ-specific sites and in blood (80, 85, 86). Multiple studies suggest Tregs delay the arrival of potential effector T cells into the pulmonary lymph nodes during early infection, play a role in reactivation of latent infection, and have a positive correlation with bacterial burden and extent of active disease (80, 81, 87, 88). Contrasting results have been observed in the TB treatment phase, with some studies reporting declining levels of Tregs and others showing a transient increase (86, 89, 90).

#### Myeloid-Derived Suppressor Cells

Myeloid-derived suppressor cells are another group of regulatory cells controlling TB inflammation. MDSCs are a diverse population of monocyte, granulocyte, and DC precursors showing features of immune suppressors (91). Similar to macrophages, polarized MDSC lineages can be distinguished as M1 and M2 cells (92). Their numbers are expanded significantly in cancer and in pleural effusions and blood of TB patients (93, 94). MDSCs have been shown to become infected by Mtb, accumulate within the inflamed lung, interact with granuloma-residing cells, and decrease in number after TB chemotherapy (95, 96).

#### Strategies for Modulation of Treg and MDSC Population

Studies of Treg suppression agents in combination with TB vaccination have yielded unclear results. A heterologous BCG/DNA-heat shock protein (HSP) 65 vaccination regimen was protective, and the effect was associated with lower numbers of Tregs in murine lungs (97). Concurrent inhibition of Th2 cells and Tregs by using the small molecule inhibitors Suplatast tosylate and D4476, respectively, during BCG vaccination improved vaccine efficacy in mice, by enhancing Mtb clearance, induction of superior Th1 responses, and long-lasting protective central memory T cell responses (98). However, reduction of Tregs using anti-CD25 antibody prior to BCG vaccination and an experimental ESAT-6 subunit TB vaccine administered concurrently with IL-28B that downregulates Tregs did not enhance the protective effect in mice models, suggesting a need to refine Treg inactivation strategies (79, 99).

Table 1 lists several other agents being tested for their regulatory cell modulation properties including damage-associated molecular patterns (DAMPs), inducible indoleamine 2,3-dioxygenase (IDO), TK, phosphodiesterase-5 (PDE5) and fatty acid oxidation (FAO), and arginase inhibitors.

Damage-associated molecular patterns (also known as alarmins) are released by dying or stressed cells and include HSPs, S100, and high mobility group box (HMGB1) proteins (111). Expansion of both Treg and MDSCs in cancer involves the participation of DAMPs (112). Interestingly, HMGB1 was evaluated as an adjuvant for a TB subunit HMGB1–ESAT-6 fusion protein vaccine in a mouse model. Poly-functional CD4 T cell-mediated immune response generated by the fusion protein vaccination correlated with protection against subsequent Mtb challenge (113). Tasquinimod, an S100A9 inhibitor currently in phase III trials for prostate carcinoma, decreased accumulation and activity of MDSCs with enhanced CD8+ responses in different combination immunotherapeutic strategies including a tumor vaccine in murine cancer models (114, 115). The potential use of DAMPs and DAMP inhibitors in TB vaccination strategies needs to be resolved.

Indoleamine 2,3-dioxygenase blocks T cell proliferation and modulates immune responses to Mtb (116–118). Increased IDO expression is strongly associated with poor outcome in many cancers and is emerging as new target for potentiation of cancer vaccines (119, 120). Similarly, reduced immunogenicity seen following MVA85A vaccination in South Africans compared to UK subjects has been attributed to increased baseline IDO activity (118). Use of IDO inhibitor drugs like epacadostat that significantly decrease Treg proliferation and increase CTL activity *in vitro* provide rationale for improving vaccine responses by combining with IDO inhibitors (121). A first-in-human, phase I clinical study, combining ipilimumab (anti CTLA-4) with a single-epitope peptide vaccine targeting IDO showed detectable T-cell responses in a subset of metastatic melanoma patients (120). New strategies targeting the IDO enzyme in cancer, either through vaccine components directed against IDO epitopes or silencing the IDO gene using small hairpin RNA plasmids, are some of the strategies with potential application in the context of TB vaccination (118, 122).

Tyrosine kinase inhibitors can also reduce the Tregs and MDSCs (123, 124), probably through multiple mechanisms, including cKIT inhibition. Sunitinib, currently used in cancer therapy, depletes MDSCs and synergized with a cancer vaccine to enhance antigen-specific immune responses and tumor eradication in mice (125). Similarly, dasatinib exhibits modest single-agent clinical efficacy but showed superior efficacy associated with reduced levels of MDSC and Treg populations in a combination treatment regimen with a DC vaccine candidate in a murine melanoma model (126). Imatinib, used as an approved therapeutic for chronic myelogenous leukemia, reduced bacterial load and associated pathology in mice infected with Mtb and acted in a synergistic manner with anti-TB drugs (47). Several mechanisms are possible for this effect, including inhibition of suppressor cells, and imatinib is being considered for study as a vaccine adjunct for infections.

### Manipulation of Immunometabolism by Mtb and Potential Interventions

#### Adenosine Monophosophate-Activated Protein Kinase

Adenosine monophosophate-activated protein kinase (AMPK) is an energy sensor kinase that regulates cellular energy homeostasis and acts as a negative regulator of inflammation (127–129). AMPK-dependent control of T cell metabolism (as an immunometabolic checkpoint) is a crucial contributing factor to determine T cell development and effector responses. AMPK also has an essential role in memory T-cell differentiation by regulating the metabolic switch from primarily aerobic glycolysis to oxidative phosphorylation of lipids (129, 130). During Mtb infection, AMPK activation enhances autophagy, leading to phagosomal maturation and improved antimicrobial response (107, 129, 131).

The antidiabetic drug metformin (MET) is an AMPK activator that inhibits intracellular Mtb growth, restricts disease immunopathology, and induces expansion of Mtb-specific effector and memory T cells (Table 1) (128). Additionally, MET-treated diabetic patients with LTBI have increased numbers of Mtb-specific T cells (128). Although issues of dosage and incorporation into current TB treatments remain, MET is under consideration for evaluation as an adjunctive agent for TB treatment (132). Therapeutic cancer vaccination combined with MET improved tumor-infiltrating lymphocytes’ multifunctionality and protection from apoptosis and exhaustion (133). Additionally, MET is able to block M2-like macrophage polarization (partially through AMPK) in cancer. The Notch signaling pathway is a potentially important mechanism in this polarization (134, 135).

##### Cross Talk of AMPK with Other Signaling Pathways, Particularly mTOR and HIF-1α

Increasing evidence indicates that T cells have metabolic checkpoints impacting their development and functions, including final phenotypes and numbers. AMPK and mTOR are chief players in the sensing and control of metabolic state and determination of subsequent immune cellular fates (136). mTOR inhibition is among the downstream effects of AMPK signaling. As a result, rapamycin, an inhibitor of mTOR, may share mechanistic effects with MET, an AMPK activator that subsequently inhibits mTOR (133).

Similarly, mTOR signaling, primarily through activating the transcription factor hypoxia-inducible factor-1 (HIF-1α), regulates T cell effector and memory differentiation (137). HIF-1α increases aerobic glycolysis (opposed by AMPK) and regulates the differentiation of CD4+ T cells, favoring differentiation into Th17 cells versus Tregs and production of pro-inflammatory cytokines in response to T cell receptor activation (136, 138). Elevated levels of HIF result in the differentiation of cytotoxic CD8+ lymphocytes (CTLs) with a high glycolytic activity, increased effector capacity, and expression of costimulatory receptors like OX40 (139). These HIF-dependent effects enhanced and sustained T cell effector function in tumor and persistent infection models (139, 140). However, HIF-1α may be a key factor for Mtb infection control, as mice lacking HIF-1α in the myeloid lineage were more susceptible to infection and exhibited defective production of inflammatory cytokines and microbicidal effectors (141). The role of HIF-1 activity in the regulation of immune responses for infections in general remains an area in need of investigation before any assessment can be made regarding potential clinical applications.

#### Sirtuins

Silent information regulator 2 proteins or sirtuins (Sirts) are seven NAD-dependent protein deacetylases involved in stress adaption, cell survival, aging, and apoptosis (142–144). Sirts, specifically Sirt1, Sirt2, Sirt6, and Sirt7, are also emerging as important players in epigenetic changes (145). Sirt1 activates the key autophagy proteins ATG5, ATG7, ATG8/LC3, and Sirt1 and Sirt2 have anti-inflammatory roles, such as inhibition of NF-kB signaling and NLRP3 inflammasome activity (146–149). Sirt1 has recently emerged as a key regulator in immune regulation modulating both innate and adaptive responses.

Sirt1 inhibitors (Table 1) are able to block virus infections including HCMV, influenza A virus, and adenovirus Ad5 (150). In a tumor microenvironment, Sirt1 deficiency impacted immune suppression by switching MDSCs to a pro-inflammatory M1 phenotype through HIF-1α-dependent glycolysis (92). This suggests that Sirt1 inhibitors should be considered for evaluation as vaccine adjuncts. Conversely, the enzymatic activity of Sirt1 can be enhanced by small molecule activators including the natural polyphenol, resveratrol, and synthetic Sirt1 activator compounds (151). In respiratory syncytial virus infection, Sirt1 promotes an effective antiviral response through DC cytokine secretion and autophagy-mediated processes, providing an option for a novel vaccination strategy, such as a Sirt1-activating viral vaccine (152).

Sirtuins have multiple effects on immune regulation based on integration of immune cell metabolic and immune function regulation including potentially modulating T cell exhaustion. Sirt1 activates PGC-1a and negatively regulates NFAT, mTOR, and HIF-1a activity (149, 153, 154). This effect could reduce metabolic cell stresses and improve the effectiveness of PD-1 blockade therapy. Overall, the role of Sirt family in Mtb infection and its potential combined use with vaccination requires further exploration.

### Immunomodulatory Potential of Core Signaling Pathways

#### Wnt/β-Catenin Signaling

The canonical Wnt/β-catenin pathway plays an important role in the regulation of various cellular processes, including cell differentiation, apoptosis, polarity, motility, embryonic development, adult tissue homeostasis, and carcinogenesis (155–158). Infection with BCG activates Wnt/β-catenin signaling, resulting in the expression of a host of genetic signatures important for subsequent regulatory responses, including cyclooxygenase (COX)-2, suppressor of cytokine signaling-3 (SOCS-3), and Jagged1 (ligand for Notch1 signaling) (159–162). Additionally, wnt/β-catenin signaling in DCs induces production of various anti-inflammatory cytokines that induce Treg cells and suppress TH1/Th17 responses (163).

The wnt/β-catenin pathway has been suggested as a therapeutic target for adjunct treatments along with vaccination in the ovarian tumor microenvironment (164). Agents for targeting the wnt/β-catenin pathway include flavonoids, monensin, and resveratrol, and the use of antibodies against the Notch receptor or ligand (Table 1) (165). Additionally, telomere protection enzymes, the tankyrase (Tnks) subfamily of PARPs, control transcriptional response to secreted Wnt signaling molecules. Tnks inhibitors are currently being developed as therapeutic agents for targeting Wnt-related cancers (166). Despite recent reports of studies implicating Wnt signaling pathway in the development and progression of TB, the utilization of potential signaling inhibitors as effective adjuncts in TB vaccination remains to be explored.

#### Notch Signaling

Notch signaling regulates various developmental stages of T and B cells and T cell activation and differentiation (167). This highly conserved pathway also integrates further inputs from other signaling pathways. This cross talk includes upregulation of ligands or receptors (e.g., Wnt regulating Notch ligand levels) and co-regulation of shared target genes [e.g., Hes1 and Gsk3β regulation by Notch, Wnt, and sonic hedgehog (SHH)] (168, 169). Notch signaling directly controls PD-1 transcription in activated CD8+ T cells, as blocking Notch signaling leads to the inhibition of PD-1 expression (170). BCG infection of murine macrophages induced upregulation of Notch1 expression. Activation of Notch1 signaling was demonstrated in granulomatous lesions in brains of humans with TB meningitis in comparison to healthy individuals (167, 171). Several notch inhibitors, mainly gamma-secretase inhibitors, are now in clinical trials, e.g., RO4929097 (Table 1) (172).

#### Sonic Hedgehog

Sonic hedgehog, a pleotropic member of the hedgehog family of signaling molecules, plays a key role in cellular homeostasis and development (173). Aberrant activation of the HH signaling pathway has been implicated in the pathogenesis of several types of cancer (174). SHH signaling constitutes one of the networks that macrophages use to tailor differential immune responses to infections with pathogenic mycobacteria. In addition to BCG triggering a robust activation of SHH signaling in macrophages, SHH signaling is heightened in humans with PTB and TBM (175). Additionally, TNFα-driven SHH signaling downregulates *M. bovis*-specific TLR2 responses through micro RNA-31 and -150 targeting MyD88 (175). These changes lead to modulation of a battery of genes that regulate various functions of macrophages genes, including SOCS-3, COX-2, MMP-9, and M1 and M2 genes and emphasize a novel role for SHH signaling in host immune responses to mycobacterial infections. Vismodegib and sonidegib are small molecule inhibitors targeting the SHH pathway in cancer (Table 1) (174, 176).

#### Glycogen Synthase Kinase-3β

The binding of Wnt ligands secreted from various cell types to transmembrane receptors of the Frizzled family activates a signaling cascade resulting in the inhibition of a negative regulator of β-catenin levels, glycogen synthase kinase-3β (GSK3β) (177). GSK3β plays a pivotal role in regulating many cellular functions, including cell survival and apoptosis (165, 178–180). *M. bovis* BCG induces phosphorylation of GSK3 by the PI3K–Akt signaling pathway, switching the transcriptional activity to an anti-inflammatory CREB-driven cytokine response instead of the pro-inflammatory NF-κB transcription (181, 182). GSK3β is an upstream kinase regulating PD-1 transcription, and the use of GSK3β inhibitors *in vivo* downregulates PD-1 and enhances CTL clearance of viral infections (183). Several inhibitors of GSK3β are in various stages of development, e.g., tideglusib (Table 1) (184). Additionally, in a DC-based tumor vaccination murine model, inhibition of GSK3β by siRNA enhanced CTL activity *via* suppression of IDO expression (185).

### Epigenetic Changes and Trained Immunity

Trained immunity (TI) is a new paradigm for vaccination referring to a prolonged, enhanced functional innate response after adequate priming. While the specificity and memory of innate immunity is not as sophisticated as adaptive immunity, TI could potentially contribute to BCG, yellow fever, influenza, vaccinia, and measles vaccine-induced responses (186). The molecular basis of innate immune memory is cell type-specific epigenetic modifications, including DNA methylation and histone modifications, such as acetylation, methylation, and phosphorylation (187). Specifically, after BCG vaccination, the TI response in human monocytes and NK cells appears to be mediated through autophagy and regulatory pathway signaling changes leading to epigenetic reprograming (188, 189). Following histone modification profiles and genome-wide transcriptomics to study trained human monocytes, an mTOR- and HIF-1α-mediated aerobic glycolysis pathway has been suggested as the metabolic basis for TI (190). The identification of glycolysis as a fundamental process in TI defines potential targets that inhibit TI including inhibition of mTOR/HIF-1a axis and use of MET and sirtuin activators (190).

Whether training of innate immunity to exhibit adaptive features can significantly potentiate vaccine responses is an important research question. Possible epigenetic-based interventions may include enhancement of TI development with sirtuin inhibition and stabilizing the activity of the mTOR/HIF axis. The knowledge that modification of epigenetic changes can potentially improve immune responses has led to development of “epi-drugs” now approved for use in targeted oncologic treatment strategies (191, 192). These drugs, inhibitors of DNA methyltransferases and histone deacetylases (HDACs) (Table 1), for example, sodium butyrate can modulate expression of immune regulatory molecules to improve immune responses in murine models, including in therapeutic combinations with cancer vaccines (193).

## Testing of Potential Agents with Candidate TB Vaccines in Appropriate Animal Models

Table 2 is a prioritized list of candidate-potentiating agents that are either approved or currently being tested for other diseases. These include activators of effector T cell expansion and generation of memory T cells responses (MET and OX40 agonists) and agents targeting specific initiators of regulatory pathway signaling (PD1, wnt/notch, and mTOR).

**Table 2:** **Prioritized list of candidate potentiating agents for use with TB vaccines**.

Target	Drug examples
**Autophagy modulation**	
Direct mechanistic target of rapamycin (mTOR) inhibitors	Sirolimus, everolimus, and ridaforolimus
mTOR independent	Imatinib
**Immune checkpoint modulation**	
Programed death-1/CTLA-1 inhibitors	Ipilimumab, pembrolizumab, and nivolumab
OX40 stimulator	OX40 agonist (monoclonal antibody)
**Immunometabolism regulation**	
Adenosine monophosophate-activated protein kinase activators	Metformin, AICAR, and AZD-769662
Sirtuins	
Activators	Resveratrol, pterostilbene, Sirt1 activator compounds in development
Inhibitors	Sirtinol and cambinol
**Core signaling pathway modulators**	
Notch inhibitors	RO4929097
Sonic hedgehog inhibitors	Vismodegib and sonidegib
Glycogen synthase kinase-3β inhibitors	Tideglusib
**Modulation of immune regulatory cells: Tregs and myeloid-derived suppressor cells**	
Damage-associated molecular patterns inhibitor	Tasquinimod
Indoleamine 2,3-dioxygenase inhibitor	Epacadostat
Tyrosine kinase inhibitors	Sunitinib, dasatinib
PDE5 inhibitors	Sildenafil

Testing the wide range of candidate TB vaccine in conjunction with immunoregulatory and immunometabolic interventions as adjuncts requires developing appropriate preclinical models. Due to the remarkable amount of cross talk between the cellular immune pathways (for example, AMPK and mTOR are chief players in the controlled sensing of metabolic state and subsequent immune cell fates), *in vitro* testing with particular focus on macrophages and DCs will be important to ascertain that specific cellular interactions are targeted without affecting other pathways. Following *in vitro* testing of new candidates, assessment of their combinatorial effect and safety interactions in small animal models including transgenic mice models and non-human primates (NHPs) will be essential evaluations. While experimental studies in mouse models have provided direct insight into Mtb- and BCG-induced changes in Wnt, Gsk3b, Notch1, SHH, and mTOR signaling (194–196), many differences in human versus murine immune cell regulation are known. In view of these differences, the use of NHPs to determine the role of regulatory pathway disruptions and the resulting effects on essential immune functions can be potentially illuminating.

As shown in the Tables 1 and 2, many of the proposed agents have been studied in the context of TB disease and are yet to be tested with candidate TB vaccines. Due to the highly complex patterns of host responses to TB infection/disease, it is probable that what relates to TB infection/disease may not be effective in the context of vaccination, and this possibility requires careful exploration. Also, differences in specific antigens and corresponding immune responses for the numerous classes of candidate TB vaccines complicate the evaluation of these adjunct agents for vaccination. Among the whole cell, viral-vectored, and adjuvanted subunit candidate vaccines, attenuated WCV may present a viable opportunity to be tested first. The antigenic complexity associated with WCV most probably will modulate key signaling pathways and/or immune cell functions that can be targeted for modification with drugs.

Timing of vaccine administration with these potential adjunct therapies remains to be determined. A balanced regulatory and effector T-cell response is required for optimal control of bacterial replication while simultaneously limiting effector cell-mediated tissue damage (197). For example, Sirt1 inhibiting drugs could potentiate vaccine responses and improve outcomes in very early stages of infection when enhanced immunity is essential. Conversely, Sirt1 activators may potentially be useful for the control of inflammatory damage and control of pathogen spread during later stages of infections. Similarly, timing of GSK3β inhibition, for instance, has to be determined as this inhibition suppresses pro-inflammatory cytokine expression, including TNF-α (198, 199). The use of inhibitors of mTOR/HIF-1a axis and MET can inhibit TI, suggesting that studies to determine the timing of use of these complementary therapies will also be needed.

## Path Forward

Tuberculosis is an ancient human disease and has very effectively adapted to persist in its hosts despite the multiple robust immune responses directed against it. While antigen-specific CD4+ T cells and macrophage-activating cytokines are required for the control of TB, merely inducing more of these T cells or cytokines may not result in improved protection (18, 200). Both Mtb and BCG bacilli are highly immunogenic and drive robust antigen-specific T cell responses (18). The limited effectiveness of BCG and failure to improve on it, underlines the lack of understanding of why highly protective immune responses are not obtained. A potential underlying cause of ineffectiveness for some Mtb vaccines may be distortion of immune cell regulatory pathways required for development of protection by many potential mechanisms.

In cancer, innovative new strategies have been developed that include restoration of proper immune checkpoint, immunometabolism and epigenetic interventions. Most of these types of interventions have much in common, as reversal of alterations will allow affected immune cells to perform necessary immune functions, including autophagy, antigen processing and presentation, cytokine secretion, and other effector mechanisms. The potential for using both immunometabolism and co-signaling interventions in combination to balance effector and regulatory effects is beginning to be explored, to potentiate host responses induced by vaccines.

Similar to cancer, the complex regulation of immune cells and the extensive cross talk among several key regulatory pathways in response to TB infection make the therapeutic targeting and utilization of immune and metabolic checkpoints in TB very challenging. The most critical need is to determine the precise points of signaling pathway dysregulation that can be reversed by targeted intervention to result in improved immune protection. A large spectrum of innovative interventions to improve immune responses are now in clinical evaluation in other diseases, including enhancement of vaccine outcomes, and are available for adaption to TB vaccine research. Truly multidisciplinary research teams including investigators in the fields of immuno-oncology, immunometabolic, and applied epigenetic research working together with more traditional pathogen vaccine researchers will be important to develop successful new TB vaccine strategies.

## Author Contributions

LJ and RH contributed equally to the development of outline and writing of the paper, reviewing relevant literature, and preparation of tables in the paper.

## Disclaimer

This paper was written by one of the authors as a National Institute of Allergy and Infectious Diseases (NIAID) employee, but the views expressed in this paper do not necessarily represent those of NIAID.

## Conflict of Interest Statement

The authors declare that the research was conducted in the absence of any commercial or financial relationships that could be construed as a potential conflict of interest.
